# ABAT gene expression associated with the sensitivity of hypomethylating agents in myelodysplastic syndrome through CXCR4/mTOR signaling

**DOI:** 10.1038/s41420-022-01170-7

**Published:** 2022-09-26

**Authors:** Guangjie Zhao, Shuang Li, Qian Wang, Wanlin Wu, Xuewei Fu, Chen Zhu, Wei Wang, Xiaoqin Wang

**Affiliations:** 1 Department of Hematology, Huashan Hospital, Fudan University, No.12 Wulumuqi Middle Road, Shanghai, China; 2 Department of Hematology, Shanghai General Hospital, Shanghai Jiao Tong University, No.85 Wujin Road, Shanghai, China

**Keywords:** Myelodysplastic syndrome, Cell signalling

## Abstract

The factors that affect hypomethylating agents (HMAs) sensitivity in myelodysplastic syndrome (MDS) are complex and multifaceted. They include DNA methylation, gene expression, mutation, etc. However, the underlying mechanisms are still not clearly illustrated. In the present work, ABAT gene expression was associated with HMAs sensitivity. It was found that ABAT gene interference increased the sensitivity of HL-60 and THP-1 cells to HMAs treatment, while ABAT overexpression decreased its sensitivity. RNA-sequencing analysis showed that ABAT knockdown activated both interferon I and interferon-gamma signaling while inhibiting the secondary metabolic synthesis and arginine metabolic process. Gas chromatography-mass spectrometry (GC-MS) based metabolic profiling also demonstrated that ABAT gene knockdown affected arginine, alanine, aspartate, and glutamate metabolism, in addition to the biosynthesis of valine, leucine, and isoleucine, and the metabolism of beta-alanine. The ABAT gene expression downregulation could activate the CXCR4/mTOR signaling pathway, which was related to HMAs sensitivity. CXCR4 expression was regulated by mTOR activity and vice versa. In vivo, mice injected with ABAT gene knockdown cells lived longer than control mice after HMAs treatment. Overall, this study elucidates the novel regulatory mechanisms of HMAs sensitivity and provides a potential therapeutic target in MDS.

## Introduction

Myelodysplastic syndrome (MDS) is a heterogeneous group of myeloid neoplasms characterized by inefficient hematopoiesis, cytopenia, and a high risk of progression to acute myeloid leukemia (AML) [1]. According to the Revised International Prognosis Scoring System (IPSS-R), the overall survival of very low-risk patients is 8.8 years, while that of very high-risk MDS patients was 0.8 years [2]. In low-risk MDS patients (IPSS-R very low, low, intermediate), therapy includes recombinant erythropoietin and red blood cell transfusion, and it is mainly aimed at improving cytopenias to prevent complications. In high-risk MDS (IPSS-R high, very high), the first-line treatment is using hypomethylating agents (HMAs) or allogeneic hematopoietic stem cell transplantation [3]. However, ~50% of patients with MDS do not respond to HMAs, and the disease eventually progresses [4]. To solve this problem, some agents were investigated in MDS to increase the sensitivity of HMAs treatment, such as BCL-2, FLT3, and IDH inhibitor, P53 activator, etc. [5, 6]. Such agents were not always found to be very efficient, and the factors that affect the efficacy of HMAs are yet to be clarified.

Some studies have shown that Tet2, DNMT3A, and IDH1/2 mutations were associated with a good response to HMAs treatment [7], while others showed that somatic mutations were not correlated with treatment response [8], and different mutation combinations may be related to HMAs response [9]. In addition, it has been found that gene expression and HMAs response are closely associated. The gene sets including TNFSF9, DBN1, ABAT, SLC24A3, MUM1, and DDX52 were highly expressed, demonstrating a good response to HMAs treatment [10]. UCK2, CAD, and DCK gene expressions that mediated the metabolism of HMAs were associated with the response [11]. mRNA expression levels in Wnt/β-catenin pathway genes also indicated the responsiveness to azacitidine treatment [12]. The ABAT gene is known to catabolize the inhibitory neurotransmitter gamma-aminobutyric acid (GABA) into succinic semialdehyde (SSA). Homozygous missense mutation of ABAT gene is related to the autosomal recessive neurometabolic disorder and mitochondrial DNA (mtDNA) depletion syndrome [13]. During normal neurodevelopment, ABAT gene expression is increased [14]. In the case of medulloblastoma, the mRNA expression level of ABAT gene was increased compared with that in the normal cerebellum. Higher ABAT expression was associated with increased GABA metabolism, which promoted cell survival in nutrient-poor conditions and induced an oxidative-phosphorylation metabolic phenotype [14]. In our previous study, we showed that high-risk MDS expressed a higher mRNA level of ABAT gene than that of low-risk MDS. The high expression level was also associated with a poor prognosis [15]. Yet, the relation of ABAT gene expression to HMAs response was not clear.

In this study, we found that ABAT gene interference increased cell apoptosis, cell differentiation and decreased the cell number of THP-1 and HL-60 cells compared to negative control after HMAs treatment. In contrast, ABAT overexpression decreased the sensitivity to HMAs treatment. It was also observed that ABAT gene knockdown induced the expression of CXCR4 gene and altered amino acid metabolism. Subsequently, the mTOR signaling pathway was activated and regulated CXCR4 expression, which played an essential role in the sensitivity of HMAs.

## Results

### Low expression of ABAT gene sensitized to HMAs treatment in THP-1 and HL-60 cells

**Fig. 1:**
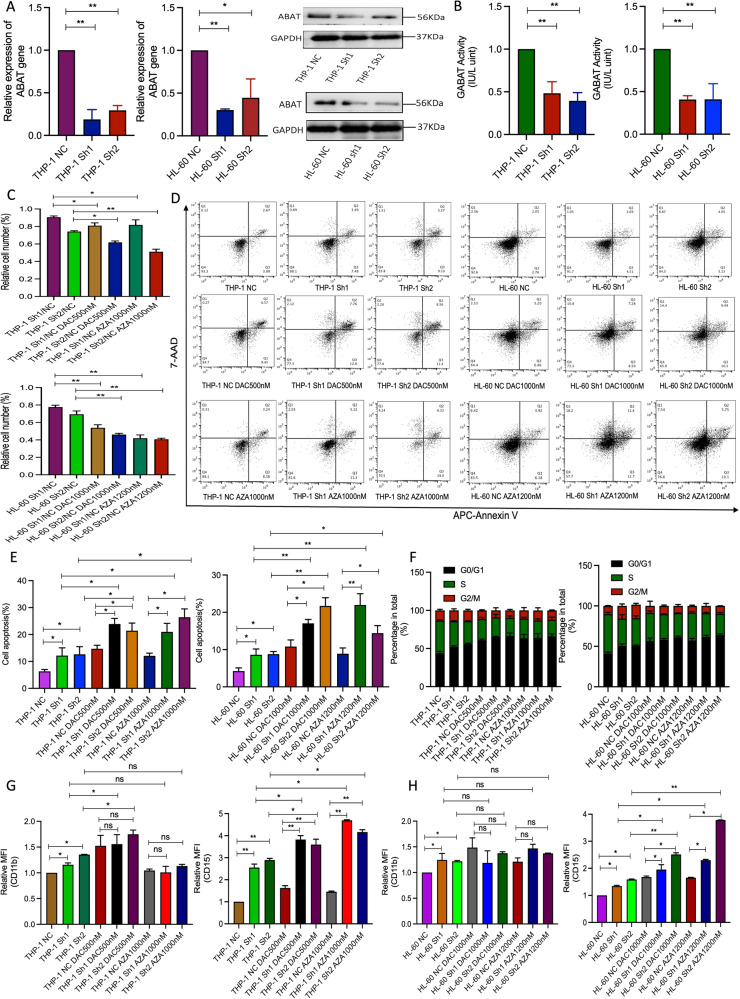
Functional analysis of THP-1 and HL-60 cells with ABAT gene knockdown and HMAs treatment. **A** mRNA and protein expression of ABAT gene in THP-1 and HL-60 cells after ABAT interference. **B** GABAT enzyme activity in both cell lines with ABAT knockdown. **C** Evaluation of relative cell number in ABAT silenced cells treated with HMAs. Representative figures (**D**) and percentage (**E**) of apoptosis in ABAT silenced cells treated with HMAs. **F** Cell cycle analysis after ABAT interference and HMAs treatment. Relative expression of CD11b and CD15 in ABAT silenced THP-1 (**G**) and HL-60 (**H**) cells with HMAs treatment. Data were represented as mean ± SEM from three biological samples. *P < 0.05, **P < 0.01. Student’s *t*-test (two groups) or one-way ANOVA analysis followed by Dunnett’s test (multiple groups). ns. not significant.

To explore the correlation between ABAT expression and HMAs sensitivity, we designed short hairpin RNA to silence ABAT gene expression. The efficacy of the Sh3 vector to downregulate ABAT gene expression was poor, so it was excluded in the following study. In THP-1 and HL-60 cells, transduction with Sh1 and Sh2 vectors significantly downregulated the mRNA and protein levels of ABAT gene compared to NC group (Fig. 1A). As a result, the enzyme activity of GABAT, which is encoded by ABAT gene, was also decreased (Fig. 1B). Subsequently, the biological cell behavior was analyzed by treating transduced cells with high concentrations of DAC and AZA for three days (because low concentration treatment did not show a significant change in cell number and apoptosis). The relative cell number was measured by dividing the cell number of ABAT-knockdown group by NC group using the same treatment. The results showed that ABAT gene interference decreased the relative cell number after HMAs treatment compared to the nontreatment group, which indicated that cells with ABAT gene interference were sensitized to HMAs treatment (Fig. 1C). In addition, cell apoptosis analysis showed that ABAT interference increased the percentage of cell apoptosis. After HMAs treatment, it increased cell apoptosis additively (Fig. 1D, E). Even though ABAT gene knockdown reduced the percentage of the S phase and prolonged G0/G1 phase, HMAs did not significantly increase the percentage of the G0/G1 phase as compared to the NC group with the same treatment (Fig. 1F). In addition, ABAT gene downregulation induced cell differentiation by upregulating the mean fluorescence intensity (MFI) of CD11b and CD15 compared to NC group. It was noticeable that after treatment of the cells with HMAs, CD15 expression was significantly upregulated as compared to NC using the same treatment. However, this treatment did not further increase the relative expression of CD11b (Fig. 1G, H). Overall, ABAT gene interference increased the sensitivity to HMAs treatment.

### ABAT overexpression decreased the sensitivity of THP-1 and HL-60 cells to HMAs treatment

**Fig. 2:**
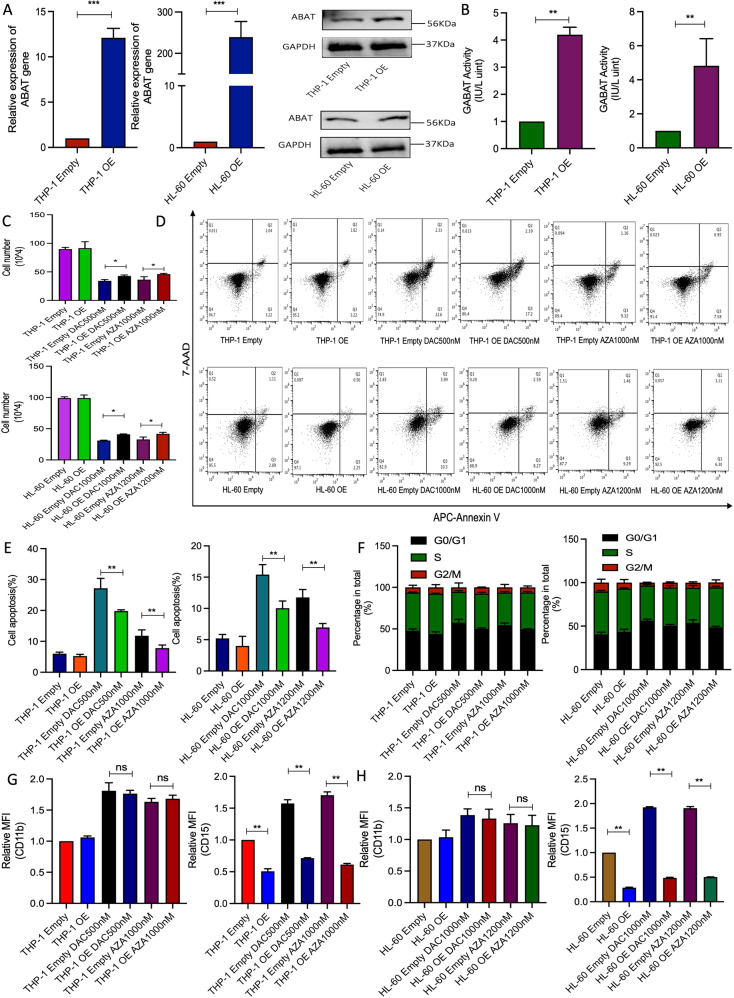
Functional analysis of THP-1 and HL-60 cells with ABAT gene overexpression and HMAs treatment. **A** mRNA and protein expression of ABAT gene in THP-1 and HL-60 cells with ABAT overexpression. **B** GABAT enzyme activity in both cell lines with ABAT overexpression. **C** Total cell number after ABAT overexpression and HMAs treatment. Representative figures (**D**) and percentage (**E**) of apoptosis in ABAT overexpressed cells treated with HMAs. **F** Cell cycle analysis after ABAT overexpression and HMAs treatment. Relative expression of CD11b and CD15 in ABAT overexpressed THP-1 (**G**) and HL-60 (**H**) cells treated with HMAs. Data were shown as mean ± SEM from three biological samples. *P < 0.05, **P < 0.01, ***P < 0.001. Student’s *t*-test (two groups) or one-way ANOVA analysis followed by Dunnett’s test (multiple groups). ns. not significant.

In our previous study, it has been demonstrated that high expression of ABAT gene resulted in a poor prognosis. Thus, similarly, the current work investigated the effect of ABAT gene overexpression in response to HMAs treatment. Results showed that relative mRNA and protein levels of ABAT gene were significantly increased compared to the empty vector group (Fig. 2A). We found higher GABAT enzyme activity after ABAT overexpression in both cell lines (Fig. 2B). Here, ABAT overexpression did not affect cell growth, apoptosis, and cell cycle, while the cell number was much higher than empty vector group after HMAs treatment (Fig. 2C). More importantly, ABAT overexpression inhibited cell apoptosis (Fig. 2D, E) and slightly decreased the percentage of the G0/G1 phase after cells were treated with HMAs (Fig. 2F). For cell differentiation, ABAT overexpression was not found to affect the expression of CD11b, but decreased the expression of CD15 significantly, even with HMAs treatment (Fig. 2G, H). Thus, it is believed that ABAT gene overexpression could play a role in decreasing the sensitivity to HMAs treatment.

### RNA-sequencing and GC-MS analysis of THP-1 cells with ABAT gene interference

**Fig. 3:**
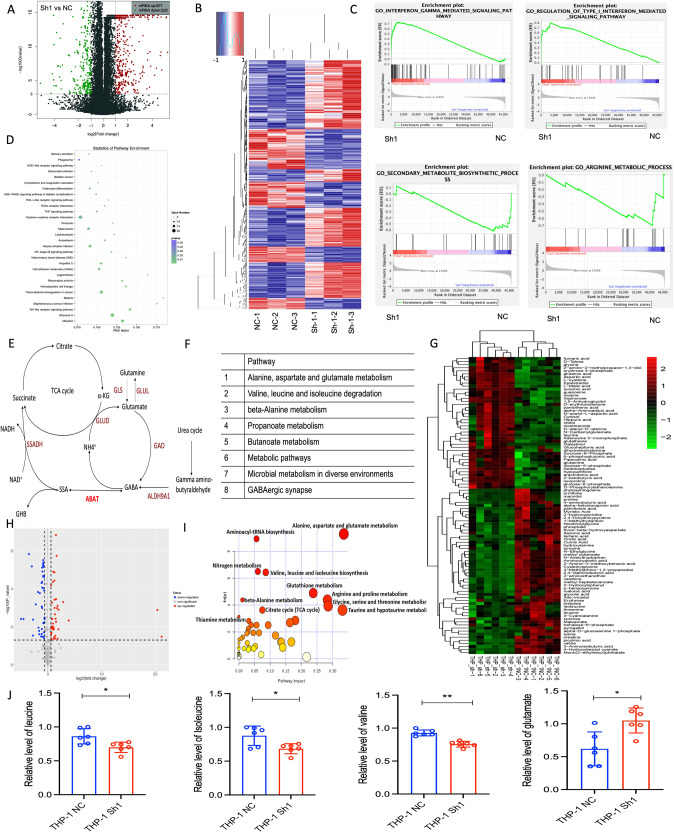
RNA-sequencing and GC-MS analysis in THP-1 cells with ABAT knockdown. **A** Volcano map of differential gene expression in THP-1 cells with ABAT interference. **B** Heatmap of differential gene expression screened in ABAT silenced THP-1 cells. **C** Gene Set Enrichment analysis of top two upregulated and downregulated pathways. **D** KEGG pathway analysis of differentially expressed genes. **E** The metabolic pathway of ABAT gene involved in disease. **F** ABAT gene-related pathway was analyzed using KEGG. **G** Heatmap of differential metabolites screened in ABAT silenced THP-1 cells. **H** Volcano map of differential metabolites in THP-1 cells with ABAT knockdown**. I** KEGG pathway analysis of differential metabolites. **J** Representative differential metabolites were analyzed between NC and ABAT knockdown groups. Data were shown as mean ± SEM from three biological samples. *P < 0.05, **P < 0.01. Student’s *t*-test was used in two-group comparisons.

To further explore the changed gene expression and metabolites after ABAT gene interference, RNA sequencing and GC-MS for THP-1 cells were performed. Compared to NC, results showed that 871 genes were upregulated while other 522 were downregulated (Fig. 3A). A profile of genes that presented differential expressions between knockdown and NC group was also determined (Fig. 3B). Subsequently, Gene Set Enrichment analysis was performed, in which upregulated pathways were found to be significantly enriched in type I interferon and interferon-gamma-mediated signaling, while downregulated pathways were observed to be enriched in secondary metabolite biosynthetic and arginine metabolic processes (Fig. 3C). KEGG analysis further showed the activation of following pathways including, measles, influenza A, toll-like receptor signaling pathway and cytokine-cytokine receptor interaction, etc. (Fig. 3D). GABAT encoded by ABAT gene catalyzes GABA into SSA, the latter partakes in TCA cycle (Fig. 3E). KEGG analysis showed that ABAT gene was related to alanine, aspartate, and glutamate metabolism, valine, leucine and isoleucine biosynthesis, and beta-alanine metabolism, etc. (Fig. 3F). In THP-1 cells, ABAT gene knockdown was responsible for the upregulation of 45 metabolites and the downregulation of 60 metabolites (Fig. 3G, H). Pathway analysis also indicated that the changed ABAT expression affected the amino acid metabolism (Fig. 3I). ABAT-knockdown THP-1 cells showed that the relative levels of valine, leucine, and isoleucine were significantly decreased, while glutamate level was increased (Fig. 3J). These results inferred that ABAT gene expression was closely related to amino acid metabolism and interferon response.

### ABAT gene interference activated CXCR/mTOR signal pathway

**Fig. 4:**
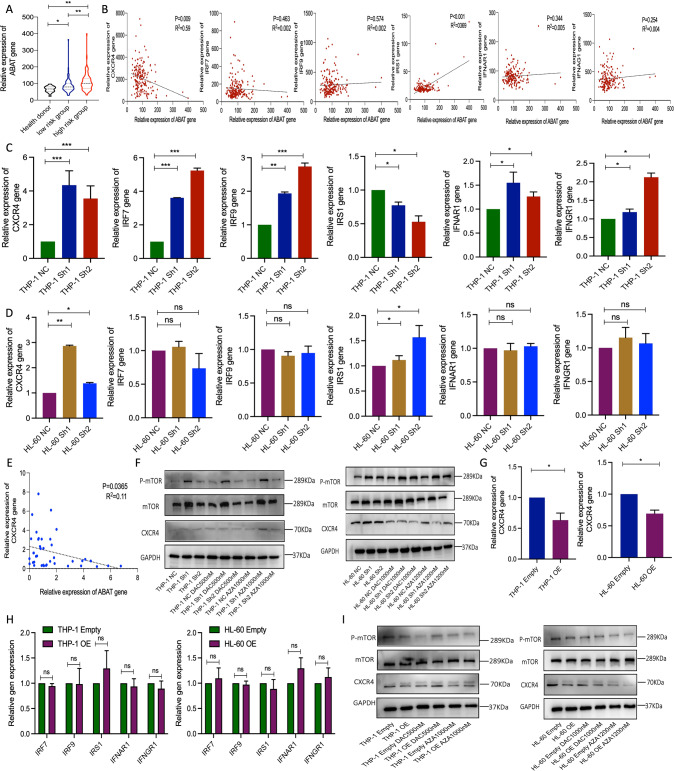
CXCR4/mTOR signaling and ABAT gene expression analysis in cell lines and clinical samples. **A** ABAT gene expression was analyzed in the GSE19429 dataset. **B** Expression correlation analysis between ABAT and interferon response-related genes in the GSE19429 dataset. Relative expression of CXCR4, IRF7, IRF9, IRS1, IFNAR1, and IFNGR1 gene in ABAT silenced THP-1 (**C**) and HL-60 (**D**) cells. **E** Correlation analysis of ABAT and CXCR4 gene in MDS samples. **F** Protein expressions of p-mTOR, mTOR, and CXCR4 in THP-1 and HL-60 cells with ABAT knockdown and HMAs treatment. **G** CXCR4 gene expression after ABAT overexpression. **H** IRF7, IRF9, IRS1, IFNAR1, and IFNGR1 gene expressions in the above two cell lines with ABAT overexpression. **I** Protein expression of p-mTOR, mTOR, and CXCR4 in THP-1 and HL-60 cells after ABAT overexpression and HMAs treatment. Data were represented as mean ± SEM from three biological samples. *P < 0.05, **P < 0.01, ***P < 0.001. Pearson correlation analysis, Student’s *t*-test (two groups), and one-way ANOVA analysis followed by Dunnett’s test (multiple groups). ns. not significant.

To find exact genes that were related to the interferon signaling after ABAT gene was silenced, the GSE19429 dataset was analyzed. It included 103 low-risk MDS patients, 80 high-risk MDS patients, and 17 healthy donors. Results of the analysis showed that ABAT gene expression was significantly higher in high-risk MDS patients (Fig. 4A). The expression correlation analysis between interferon-related genes and ABAT showed a negative correlation with the ABAT gene for CXCR4 gene expression only (Fig. 4B). In THP-1 and HL-60 cells, CXCR4 gene showed the same expression trend after ABAT gene was silenced (Fig. 4C, D). A negative expression correlation between CXCR4 and ABAT gene was also found in forty MDS patients (Fig. 4E). More importantly, since mTOR signaling is the direct sensor of amino acid level, it was inferred that ABAT gene expression may also affect mTOR activation. The results showed that ABAT gene knockdown activated mTOR signaling (Fig. 4F). However, ABAT gene overexpression downregulated CXCR4 gene expression (Fig. 4G, H) and inhibited the activation of mTOR pathway (Fig. 4I).

**Fig. 5:**
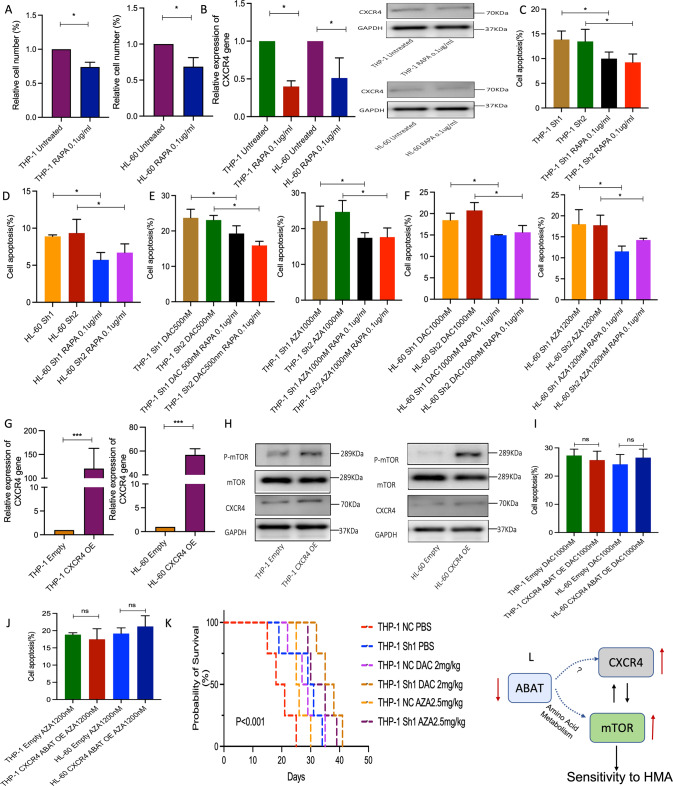
CXCR4/mTOR regulation demonstrated in vitro and prognosis analysis of ABAT gene expression in vivo. **A** Cell number in THP-1 and HL-60 cells treated with RAPA. **B** Relative expression of CXCR4 gene in cells with RAPA treatment. Cell apoptosis in ABAT silenced THP-1 (**C**) and HL-60 (**D**) cells treated with RAPA. Percentage of apoptosis in ABAT silenced THP-1 (**E**) and HL-60 (**F**) cells after RAPA and HMAs treatment. **G** CXCR4 overexpression in THP-1 and HL-60 cells. **H** Protein expression of p-mTOR, mTOR, and CXCR4 in the above two cell lines with CXCR4 overexpression. Percentage of apoptosis in cells with ABAT and CXCR4 overexpression and DAC (**I**) and AZA (**J**) treatment. **K** The survival of NOD/SCID mice injected with ABAT silenced THP-1 cells and HMAs treatment (log-rank test). **L** Schematic of ABAT/CXCR4/mTOR regulation and sensitivity to HMAs. Data were represented as mean ± SEM from three biological samples. *P < 0.05, **P < 0.01, ***P < 0.001. Student’s *t*-test (two groups) and one-way ANOVA analysis followed by Dunnett’s test (multiple groups). ns. not significant.

To investigate the regulation of ABAT gene to CXCR4/mTOR signaling, Rapamycin (RAPA) was used to treat both cell lines. Similar to previous findings, the results herein showed that RAPA could inhibit cell growth without affecting cell apoptosis (Fig. 5A). RAPA significantly downregulated the mRNA and protein level of CXCR4 gene (Fig. 5B) while also decreasing the percentage of cell apoptosis in ABAT silenced cells, with or without HMAs treatment (Fig. 5C-F). In THP-1 and HL-60 cells, CXCR4 overexpression activated the mTOR pathway (Fig. 5G, H). In ABAT gene overexpressed THP-1 and HL-60 cells, CXCR4 overexpression blocked the inhibition of cell apoptosis caused by ABAT overexpression when they were treated with HMAs (Fig. 5I, J). More Importantly, NOD/SCID mice injected with ABAT silenced THP-1 cells survived longer than NC group with or without HMAs treatment (Fig. 5K). Thus, ABAT gene expression could affect HMAs sensitivity through CXCR4/mTOR signaling.

## Discussion

Gene expression was closely related to the pathogenesis of MDS, such as that of MSI2, BMI1, SETBP1, BAALC, FLT3, MN1, and ERG gene [16]. Gene expression in signal pathways, such as thrombopoietin, IL-3, and natural killer cell signaling, was also upregulated in MDS [17]. Through analyzing the GSE19429 dataset, higher expression of ABAT gene was found in high-risk MDS compared to low-risk MDS and healthy donors. In our previous research, higher ABAT gene expression was associated with poor prognosis in MDS [15]. Neman J et al. reported that ABAT gene was more highly expressed in breast cancer metastases than in the brain [18]. In estrogen receptor-positive (ER+) and negative (ER−) breast cancer, low expression of ABAT gene is found to be related to poor prognosis [19]. Importantly, decreased expression of ABAT gene hallmarked ER-positive inflammatory breast cancer and endocrine therapy resistance in advanced disease [20]. In this study, ABAT gene knockdown increased the sensitivity of THP-1 and HL-60 cells to HMAs treatment, while ABAT gene overexpression decreased HMAs sensitivity and inhibited cell differentiation. Thus, it indicated that the role of ABAT gene in different diseases is varied.

GABAT, encoded by the ABAT gene, is mainly implicated in the GABA transamination process and was found to play an important role in the GABA shunt pathway [21]. The present work investigated the relationship between enzyme activity of GABAT and HMAs sensitivity. However, it was found that no enzyme activity inhibition could sensitize cells to HMAs treatment (data not shown). So, the non-enzyme activity of GABAT may play a key role in affecting HMAs sensitivity. While research showed that GABAT interacted with SUCLG1, SUCLG2, SUCLA2, and NDPK, and it is also involved in the mitochondrial nucleoside salvage pathway in the conversion of dNDP to dNTP through enzyme activity [13]. In breast cancer, ABAT protein and mRNA expression showed a strong negative correlation with beta-alanine abundance [22]. The results of the present work indicated that ABAT gene expression was related to amino acid metabolism. It was inferred that different diseases may harbor different metabolites preferentially. Further investigation is still required to determine the interaction of GABAT protein in MDS and the effect of ABAT gene expression on the amino acid levels.

mTOR is a conserved regulator that couples amino acid availability to cell growth, proliferation, and survival [23, 24]. In addition, the activation of PI3K-AKT-mTOR signaling pathway could also promote necrotic cell death [25]. mTORC1 activation by glutaminolysis during nutritional imbalance inhibited autophagy and induced apoptosis in cancer cells [26]. In hepatocellular carcinoma, knockdown of BCAT1 or administration of leucine activated mTOR signaling, inhibited autophagy, and increased cisplatin sensitivity and percentage of cell apoptosis [27]. The present work found that ABAT gene interference increased the phosphorylation level of mTOR and induced cell death, while ABAT gene overexpression inhibited the activation of mTOR signaling. The RNA-sequencing analysis demonstrated that ABAT gene knockdown activated interferon response. By analyzing the GEO dataset and MDS samples, a negative expression correlation between ABAT gene and CXCR4 was found. The way that ABAT gene expression influences CXCR4 pathway deserves further investigation. More importantly, CXCR4 directly controlled cell proliferation of non-hematopoietic cells and tumor cell growth and transduced signaling through mTOR pathway [28, 29]. In AML cells, CXCR4 chemokine receptor signaling induced cell death via regulation of BCL-2 family members Bcl-XL, Bak, and Noxa [30]. In this study, CXCR4 upregulation activated mTOR pathway, and mTOR inhibition decreased CXCR4 expression in THP-1 and HL-60 cells. More specifically, RAPA inhibited cell apoptosis after HMAs treatment in cells with ABAT interference. In addition, CXCR4 overexpression blocked the inhibition of cell apoptosis caused by ABAT overexpression. Those results showed that ABAT gene expression directly influenced the activation of CXCR4/mTOR pathway.

In conclusion, the findings of this study showed that ABAT gene expression was closely related to HMAs sensitivity and was involved in amino acid metabolism. ABAT gene knockdown upregulated CXCR4 expression and activated mTOR signaling, and finally promoted cell death (Fig. 5L). Therefore, targeting CXCR4/mTOR signaling pathway or ABAT is a potential therapeutic target to enhance HMAs sensitivity.

## Methods

### Patients and bone marrow samples

Bone marrow (BM) samples were extracted from 40 adult patients with MDS, of which 22 were men and 18 were women. According to the 2016 World Health Organization criteria, the diagnosis of MDS was made and revised [31], among which 11 had MDS with an excess of blasts type 1 (MDS-EB‑1), 8 had MDS-EB-2, 7 had MDS with multilineage dysplasia, 9 had MDS with single lineage dysplasia, and 5 had MDS with ring sideroblasts. The median age was 56 years old. All BM samples were obtained between December 30, 2015, and January 1, 2018. This study was approved by the Ethics Committee of Huashan Hospital, Fudan University (Approval No. KY2015‑269; Shanghai, China), and informed consent was obtained from each participant.

### RNA isolation and quantitative reverse transcription PCR

The bone marrow mononuclear cells (BMNC) were isolated by a lymphocyte separation medium (Ficoll® Paque Plus; GE Healthcare, Chicago, IL, USA) according to the manufacturer’s protocol. The TRIzol® reagent (Invitrogen; Thermo Fisher Scientific, Inc., Waltham, MA, USA) was used to extract RNA from BMNC and cultured leukemia cells. The RNA was reverse transcribed into cDNA using Hifair® III 1st Strand cDNA Synthesis SuperMix for qPCR (gDNA digester plus) (Yisheng, lnc., Shanghai, China). For the real-time PCR, cDNA samples were amplified using CFX96 Touch Real-Time PCR Detection System (Bio-Rad Laboratories, Inc. California, USA) with Hieff UNICON® qPCR SYBR Green Master Mix (Yisheng, lnc., Shanghai, China). The sequences of PCR primers are described in Supplementary Table 1. The relative expression level of the target gene was normalized to GAPDH using the 2-ΔΔCq (fold change) method.

### Cell culture

In this study, the human myeloid leukemia cell lines THP‑1 and HL-60 were obtained from the Chinese Academy of Sciences (Shanghai, China). All cells were cultured in RPMI-1640 (HyClone; GE Healthcare Life Sciences, Logan, UT, USA) at 37 °C in Heracell VIOS 160i Incubator (Thermo Fisher Scientific, Inc., Waltham, MA, USA) containing 5% CO_2_ and were supplemented with 10% fetal bovine serum (Gemini; Gemini Bio lnc., Woodland, USA).

### Cell transfection

For ABAT gene interference, three target oligonucleotide sequences were synthesized and cloned into pGMLV‑SC5 lentiviral vector (Genomeditech, Shanghai, China). The three sequences were: 5′-GCGGGAGGACCTGCTAA ATAA-3′ (Sh1); 5′‑GCTGGAGACGTGCATGATTAA-3′ (Sh2); and 5′‑GGTGAC AAATCCATTCGTTTC‑3′ (Sh3). The pGMLV‑SC5 vector was used as a negative control (NC). The cDNA sequence of ABAT and CXCR4 gene was cloned into PGMLV-CMV-EF1-ZsGreen1-T2A-Puro plasmid (Genomedi-tech, Shanghai, China). To prepare the lentivirus in 293 T cells, the viral packaging plasmid (psPAX2) and viral envelope plasmid (pMD2G) were used, and the virus was concentrated by lentivirus concentration solution (Yisheng, lnc., Shanghai, China).

### Drug treatment

For each cell line, 2 × 10^5^ cells were seeded in 12 wells and transduced with lentivirus, as previously mentioned. Additionally, they were treated with 5-Aza-2’-deoxycytidine (DAC, Sigma–Aldrich; Merck KGaA, Darmstadt, Germany) and 5-Azacytidine (AZA, Sigma–Aldrich) at concentrations of 500 nM and 1000, 1000, and 1200 nM based on IC50 of each cell line, respectively. For combined drug treatment, a medium concentration of rapamycin was used (RAPA, Sigma–Aldrich). The two cell lines were thereafter treated for 72 h by incubation with DAC, AZA, or combined RAPA with DAC or AZA. The GABAT enzyme activity was measured using the GABAT assay kit (Biomedical Research Service Center, University at Buffalo, Buffalo, NY, USA) following the protocol.

### Cell cycle, cell apoptosis, and cell differentiation

Cell growth was analyzed at 72 h by counting total cell numbers using trypan blue solution (Thermo Fisher Scientific, Inc., Waltham, MA, USA). For cell cycle analysis, the cells were fixed using the Cytofix/Cytoperm™ Fixation/Permeabilization Solution Kit (BD Biosciences, NJ, USA) and were incubated with propidium iodide solution. Apoptotic fractions were analyzed by Annexin V/7-AAD staining using the APC Annexin V Apoptosis Detection Kit I (BioLegend, BioLegend, Inc., San Diego, CA, USA). Cell lineage-specific marker expression was measured by flow cytometry using CD15-PE, CD11b-APC, and 7-AAD antibodies (BioLegend, Inc., San Diego, CA, USA).

### RNA sequencing

For library preparation, the TruSeq RNA Sample Prep Kit v2 (Illumina, Inc., San Diego, CA, USA) was used. Briefly, 500 ng of total mRNA was poly-A purified and fragmented. Then, first-strand cDNA was synthesized using random primers. Subsequently, second-strand cDNA was synthesized and end-repaired, to which a single A base and adaptor ligation were added. PCR amplification was then performed, and the enriched cDNA libraries were sequenced using the Illumina HiSeq 2000 at the Ribobio High throughput sequencing station (Ribobio, lnc., Guangzhou, China). Genomic and split-mapped reads were used to quantify the gene expression level and were provided as RPKM (Reads Per Kilobase per Million mapped reads) values, which were converted to log2 scale. Statistical significance was considered for “q-value” less than 0.05, whereas an expression fold change greater than two-fold was regarded as significant.

### Gas chromatography-mass spectrometry (GC-MS)

The GC-MS analysis was conducted using an Agilent Gas chromatography-mass spectrometer (Agilent, 7890A-5975C, CA, USA). The column used for reversed-phase separation was a HP-5MS column (30 m × 0.25 mm × 0.25 μm) (Agilent J&W Scientific, Folsom, CA, USA). The initial temperature was 60 °C and was increased to 310 °C at an 8 °C/min gradient, which was then maintained for 6 min. The metabolites were separated at 36 min intervals at a flow rate of 1 mL/min. The mass spectrometer was operated in positive ion mode using the *m/z* range of 50–60 *m/z*. ChromaTOF software (v 4.34, LECO, St Joseph, MI) was used to analyze the original data. Subsequently, differential metabolites identification was performed by Orthogonal Projections to Latent Structures Discriminant Analysis (OPLS-DA).

### Western blot analysis

Total protein was extracted from the cultured cells using protein lysis buffer (Thermo Fisher Scientific, Inc.) and was quantified using PierceTM BCA protein assay kit (Thermo Fisher Scientific, Inc). The Protein samples (20 μg) were fractionated by 10% SDS‑PAGE or 6% SDS‑PAGE, after which they were transferred onto polyvinylidene fluoride membranes (Bio‑Rad Laboratories, Inc.). The membranes were immune-stained overnight at 4 °C using antibodies against ABAT (Cat.ab216465, 1:1000; Abcam, Cambridge, MA, USA), CXCR4 (Cat.11073-2-AP,1:1000, Proteintech, Manchester, United Kingdom), mTOR (Cat.66888-1-Ig, 1:1000, Proteintech), p-mTOR (Cat.67778-1-Ig, 1:1000, Proteintech). GAPDH (Cat.60004-1-Ig 1:1000, Proteintech) antibody was used as loading controls. After washing with Tris‑buffered saline (TBS) containing 0.1% Tween, the membranes were incubated at room temperature for 1 h with the following secondary antibodies: Anti‑rabbit immunoglobulin G (IgG) (Cat.7074P2, 1:3000; Cell Signaling Technology, Inc.) and anti‑mouse IgG (Cat.ab6728, 1:3000; Abcam). The protein bands were visualized using the Fuji LAS 3000 system.

#### Mice

A half-million GFP-positive THP-1 cells were intravenously injected into eight-week-old male NOD/SCID mice after Busulfan treatment (25 mg/Kg. Sigma–Aldrich) for 48 h. For experiments that required drug administration, the thirty recipient mice were randomly assigned to distinct treatment groups (five in each group) and treated with Phosphate Buffered Saline (PBS), DAC, or AZA daily by intraperitoneal injection. The procedure was performed on day 7, for 3 and 5 consecutive days, respectively. Peripheral blood of recipient NOD/SCID mice was collected, and flow cytometry was used after 11 days to analyze GFP-positive cells. The overall survival of mice was decorated. The experiments were conducted in accordance with the care and use of laboratory animals and approved by The Fudan University Animal Ethics Committee.

### Statistical analysis

Data were expressed as mean ± standard deviation and analyzed using GraphPad Prism 8.0. Comparison between two groups was analyzed by student’s *t*‑test. For multiple group comparison, one-way ANOVA followed by Dunnett’s test was performed. In addition, the survival comparison of different mice groups was measured by Kaplan–Meier analysis and log-rank test. Pearson correlation analysis was also used to analyze the relevance. *P* < 0.05 was considered statistically significant. The following annotations were used, *P < 0.05, **P < 0.01, and ***P < 0.001.

## Supplementary information

Supplementary Table

Supplementary Original WB

## Data Availability

The original RNA-seq data are available on NCBI. SRA data, BioProject No. PRJNA855444. Further inquiries can be directed to the corresponding author.

## References

[1] Zeidan AM, Shallis RM, Wang R, Davidoff A, Ma X (2019). Epidemiology of myelodysplastic syndromes: why characterizing the beast is a prerequisite to taming it. Blood Rev.

[2] Chen-Liang TH (2021). Prognosis in myelodysplastic syndromes: the clinical challenge of genomic integration. J Clin Med.

[3] Platzbecker U (2019). Treatment of MDS. Blood.

[4] Fenaux P, Mufti GJ, Hellstrom-Lindberg E, Santini V, Finelli C, Giagounidis A (2009). Efficacy of azacitidine compared with that of conventional care regimens in the treatment of higher-risk myelodysplastic syndromes: a randomised, open-label, phase III study. Lancet Oncol.

[5] Stomper J, Rotondo JC, Greve G, Lübbert M (2021). Hypomethylating agents (HMA) for the treatment of acute myeloid leukemia and myelodysplastic syndromes: mechanisms of resistance and novel HMA-based therapies. Leukemia.

[6] Zhao G, Wang Q, Li S, Wang X (2021). Resistance to hypomethylating agents in myelodysplastic syndrome and acute myeloid leukemia from clinical data and molecular mechanism. Front Oncol.

[7] Traina F, Visconte V, Elson P, Tabarroki A, Jankowska AM, Hasrouni E (2014). Impact of molecular mutations on treatment response to DNMT inhibitors in myelodysplasia and related neoplasms. Leukemia.

[8] Meldi K, Qin T, Buchi F, Droin N, Sotzen J, Micol JB (2015). Specific molecular signatures predict decitabine response in chronic myelomonocytic leukemia. J Clin Invest.

[9] Nazha A, Sekeres MA, Bejar R, Rauh MJ, Othus M, Komrokji RS, et al. Genomic biomarkers to predict resistance to hypomethylating agents in patients with myelodysplastic syndromes using artificial intelligence. JCO Precis Oncol. 2019;3:PO. 19.00119. 10.1200/PO.19.00119 PMC6818517 31663066

[10] Bohl SR, Claus R, Dolnik A, Schlenk RF, Döhner K, Hackanson B (2013). Decitabine response associated gene expression patterns in acute myeloid leukemia (AML). Blood..

[11] Gu X, Tohme R, Tomlinson B, Sakre N, Hasipek M, Durkin L (2021). Decitabine- and 5-azacytidine resistance emerges from adaptive responses of the pyrimidine metabolism network. Leukemia..

[12] Kim K, Park S, Choi H, Kim HJ, Kwon YR, Ryu D (2020). Gene expression signatures associated with sensitivity to azacitidine in myelodysplastic syndromes. Sci Rep.

[13] Besse A, Wu P, Bruni F, Donti T, Graham BH, Craigen WJ (2015). The GABA transaminase, ABAT, is essential for mitochondrial nucleoside metabolism. Cell Metab.

[14] Martirosian V, Deshpande K, Zhou H, Shen K, Smith K, Northcott P (2021). Medulloblastoma uses GABA transaminase to survive in the cerebrospinal fluid microenvironment and promote leptomeningeal dissemination. Cell Rep.

[15] Zhao G, Li N, Li S, Wu W, Wang X, Gu J (2019). High methylation of the 4-aminobutyrate aminotransferase gene predicts a poor prognosis in patients with myelodysplastic syndrome. Int J Oncol.

[16] Shiozawa Y, Malcovati L, Gallì A, Pellagatti A, Karimi M, Sato-Otsubo A (2017). Gene expression and risk of leukemic transformation in myelodysplasia. Blood.

[17] Pellagatti A, Cazzola M, Giagounidis A, Perry J, Malcovati L, Della Porta MG (2010). Deregulated gene expression pathways in myelodysplastic syndrome hematopoietic stem cells. Leukemia.

[18] Neman J, Termini J, Wilczynski S, Vaidehi N, Choy C, Kowolik CM (2014). Human breast cancer metastases to the brain display GABAergic properties in the neural niche. Proc Natl Acad Sci USA.

[19] Cardoso MR, Santos JC, Ribeiro ML, Talarico MCR, Viana LR, Derchain SFM (2018). A metabolomic approach to predict breast cancer behavior and chemotherapy response. Int J Mol Sci.

[20] Jansen MP, Sas L, Sieuwerts AM, Van Cauwenberghe C, Ramirez-Ardila D, Look M (2015). Decreased expression of ABAT and STC2 hallmarks ER-positive inflammatory breast cancer and endocrine therapy resistance in advanced disease. Mol Oncol.

[21] Knott EL, Leidenheimer NJ (2020). A targeted bioinformatics assessment of adrenocortical carcinoma reveals prognostic implications of GABA system gene expression. Int J Mol Sci.

[22] Budczies J, Brockmöller SF, Müller BM, Barupal DK, Richter-Ehrenstein C, Kleine-Tebbe A (2013). Comparative metabolomics of estrogen receptor positive and estrogen receptor negative breast cancer: alterations in glutamine and beta-alanine metabolism. J Proteom.

[23] Hung CM, Garcia-Haro L, Sparks CA, Guertin DA (2012). mTOR-dependent cell survival mechanisms. Cold Spring Harb Perspect Biol.

[24] Jewell JL, Russell RC, Guan KL (2013). Amino acid signalling upstream of mTOR. Nat Rev Mol Cell Biol.

[25] Wu YT, Tan HL, Huang Q, Ong CN, Shen HM (2009). Activation of the PI3K-Akt-mTOR signaling pathway promotes necrotic cell death via suppression of autophagy. Autophagy.

[26] Villar VH, Nguyen TL, Delcroix V, Terés S, Bouchecareilh M, Salin B (2017). mTORC1 inhibition in cancer cells protects from glutaminolysis-mediated apoptosis during nutrient limitation. Nat Commun.

[27] Luo L, Sun W, Zhu W, Li S, Zhang W, Xu X (2021). BCAT1 decreases the sensitivity of cancer cells to cisplatin by regulating mTOR-mediated autophagy via branched-chain amino acid metabolism. Cell Death Dis.

[28] Bianchi ME, Mezzapelle R (2020). The chemokine receptor CXCR4 in cell proliferation and tissue regeneration. Front Immunol.

[29] Ieranò C, Santagata S, Napolitano M, Guardia F, Grimaldi A, Antignani E (2014). CXCR4 and CXCR7 transduce through mTOR in human renal cancer cells. Cell Death Dis.

[30] Kremer KN, Peterson KL, Schneider PA, Meng XW, Dai H, Hess AD (2013). CXCR4 chemokine receptor signaling induces apoptosis in acute myeloid leukemia cells via regulation of the Bcl-2 family members Bcl-XL, Noxa, and Bak. J Biol Chem.

[31] Hong M, He G (2017). The 2016 Revision to the World Health Organization Classification of Myelodysplastic Syndromes. J Transl Int Med.

