# Supplementary material for: ABAT gene expression associated with the sensitivity of hypomethylating agents in myelodysplastic syndrome through CXCR4/mTOR signaling
Source: Cell Death Discov. 2022 Sep 26;8:398. doi: 10.1038/s41420-022-01170-7 (PMC9512903; doi:10.1038/s41420-022-01170-7)
Supplement: Supplementary file 1 — Supplementary Table [file 41420_2022_1170_MOESM1_ESM.docx]

**Table 1. The sequences of primers for target genes.**

| Gene | Sequences | Gene | Sequences |
| --- | --- | --- | --- |
| ABAT | F: CCGACTACAGCATCCTCTCC  R: GGTTCTCTTTCACAAACTCTTCC | IRS1 | F: AGGTGGATGA CTCTGTGGTG  R: GGGATTGTTGAGATGGTGCC |
| GAPDH | F: AGAAGGCTGGGGCTCATT TG  R: AGGGGCCATCCACAGTCTTC | CXCR4 | F: CATCAGTCTGGACCGCTACC  R: GGCAGGATAAGGCCAACCAT |
| IFNAR1 | F: CAGTGGCTCCACGCCTTTTTA  R: TCAGA TGCTTGTACGCGGAG |  |  |
| IFNGR1 | F: AGTGCTTAGCCTGGTATTCATCTG  R: GGCTGGTATGACGTGATGAGTG |  |  |
| IRF7 | F: CCACGCTATACCATCTACCTGG  R: GCTGCTATCCAGGGAAGACACA |  |  |
| IRF9 | F: TTCTGTCCCTGGTGTAGAGCCT  R: TTTCAGGACACGATTATCACGG |  |  |
