# Supplementary material for: ABAT gene expression associated with the sensitivity of hypomethylating agents in myelodysplastic syndrome through CXCR4/mTOR signaling
Source: Cell Death Discov. 2022 Sep 26;8:398. doi: 10.1038/s41420-022-01170-7 (PMC9512903; doi:10.1038/s41420-022-01170-7)

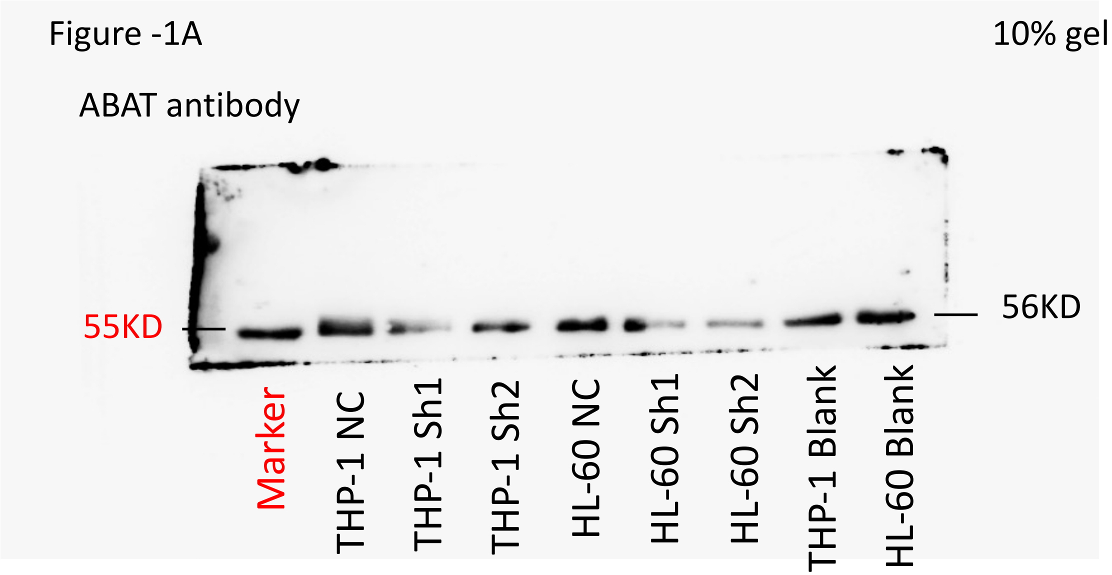
Figure -1A
10% gel
ABAT antibody
55KD
56KD
Marker
THP-1 NC
THP-1 Sh1
THP-1 Sh2
HL-60 NC
HL-60 Sh1
HL-60 Sh2
THP-1 Blank
HL-60 Blank

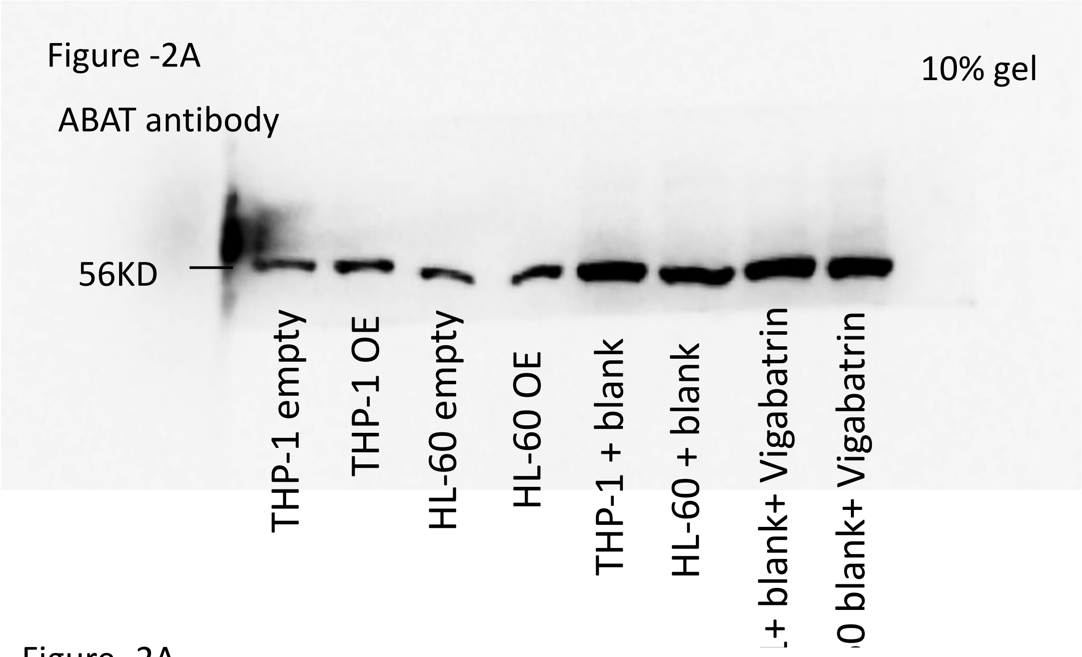
Figure -2A
10% gel
ABAT antibody
56KD
THP-1 empty
THP-1 OE
HL-60 empty
HL-60 OE
THP-1 + blank
HL-60 + blank
+ blank+ Vigabatrin
0 blank+ Vigabatrin

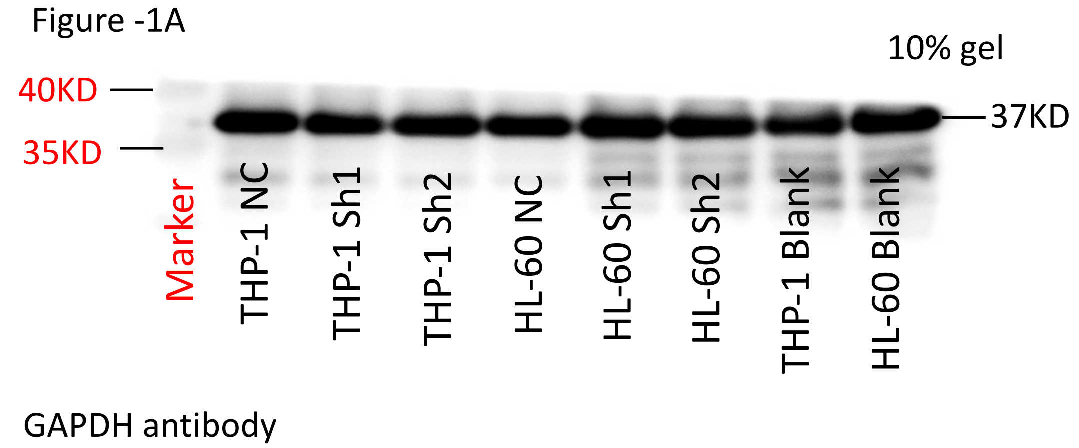
Figure -1A
10% gel
40KD
35KD
37KD
Marker
THP-1 NC
THP-1 Sh1
THP-1 Sh2
HL-60 NC
HL-60 Sh1
HL-60 Sh2
THP-1 Blank
HL-60 Blank
GAPDH antibody

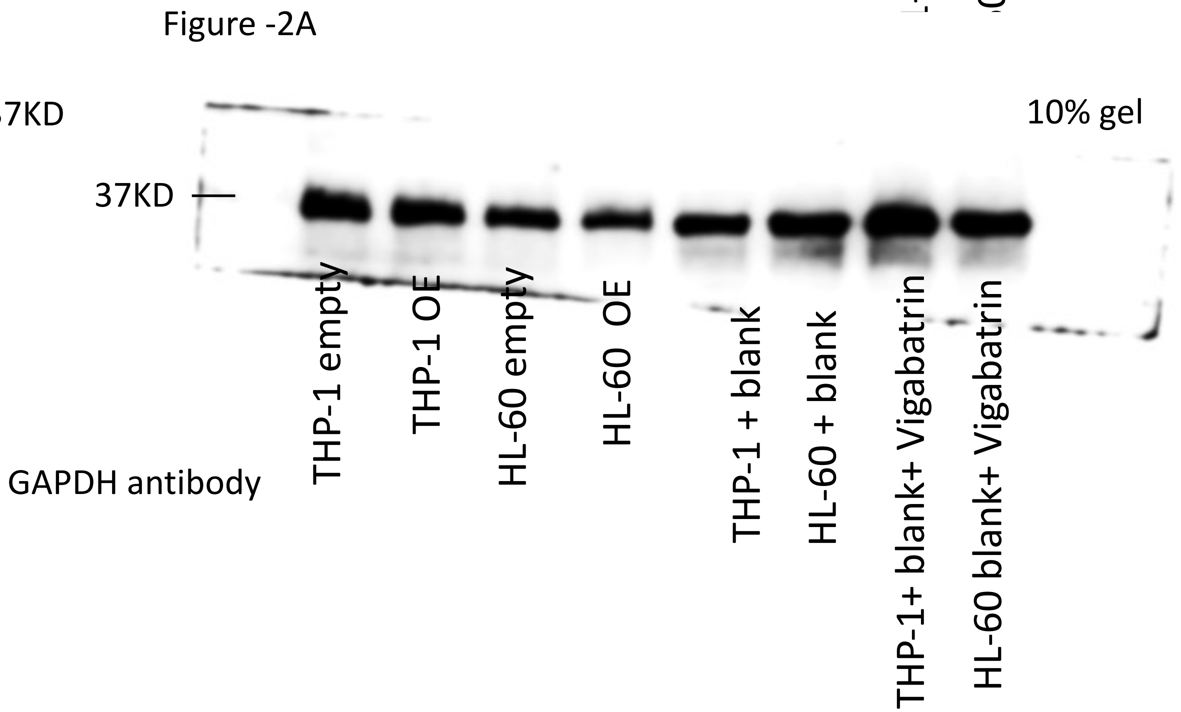
Figure -2A
10% gel
37KD
THP-1 empty
THP-1 OE
HL-60 empty
HL-60 OE
THP-1 + blank
HL-60 + blank
THP-1+ blank+ Vigabatrin
HL-60 blank+ Vigabatrin
GAPDH antibody

Figure -4F

mTOR antibody

6% gel

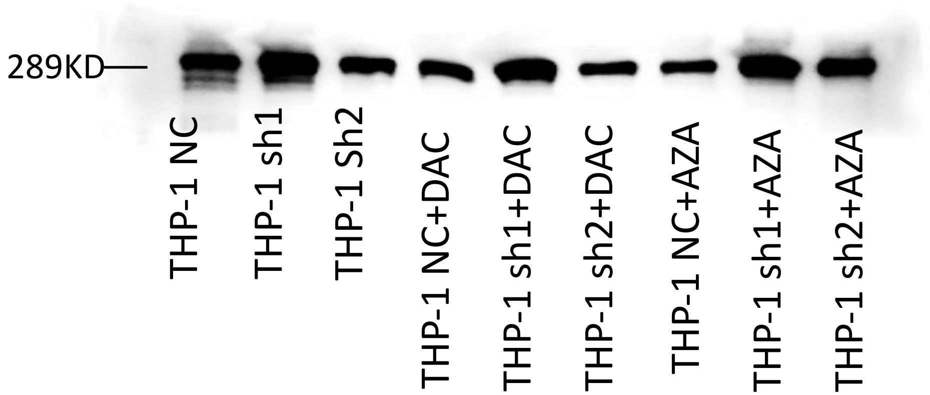

CXCR4 antibody

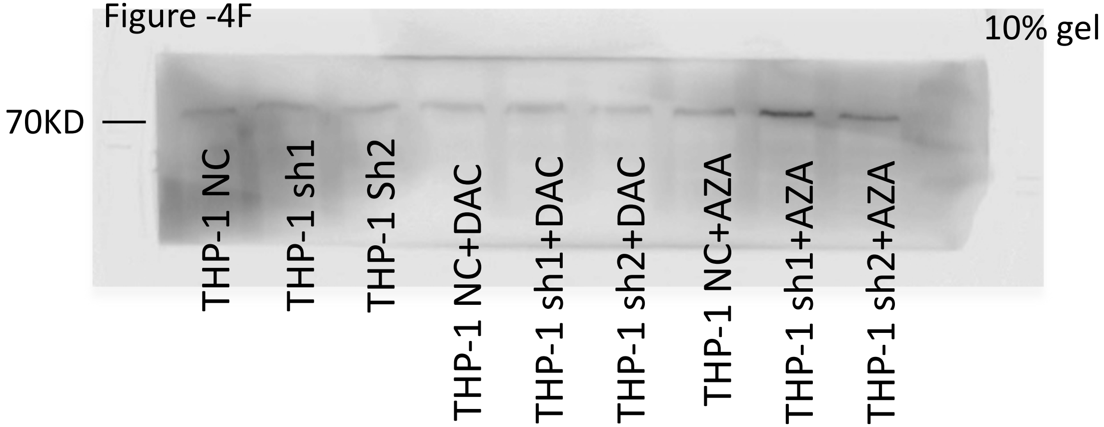

Figure -4F

6% gel

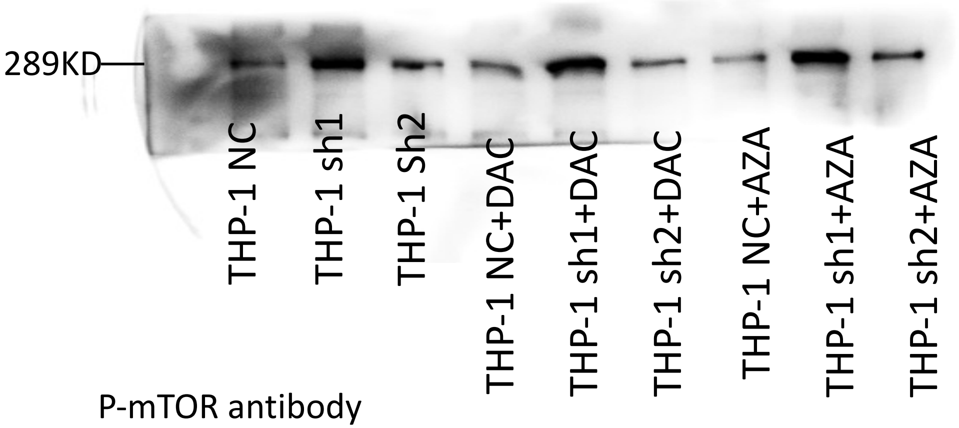

P-mTOR antibody

Figure -4F

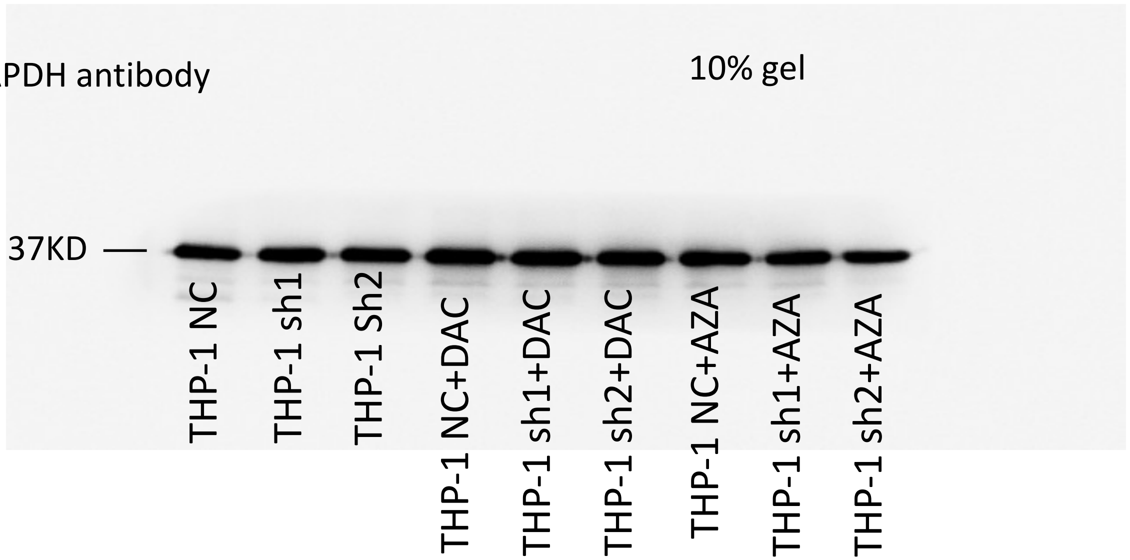

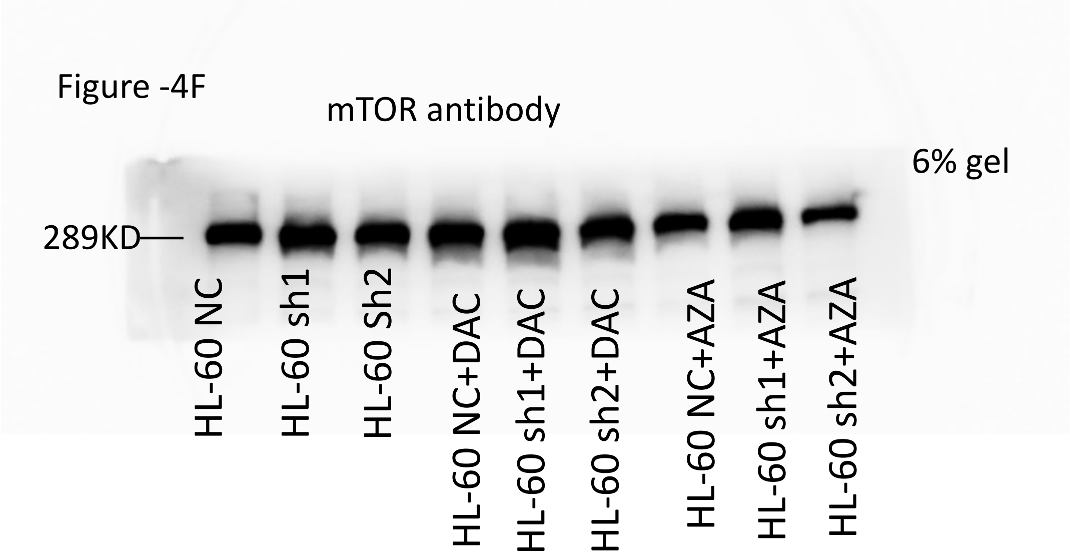
Figure -4F
mTOR antibody
6% gel
289KD
HL-60 NC
HL-60 sh1
HL-60 Sh2
HL-60 NC+DAC
HL-60 sh1+DAC
HL-60 sh2+DAC
HL-60 NC+AZA
HL-60 sh1+AZA
HL-60 sh2+AZA

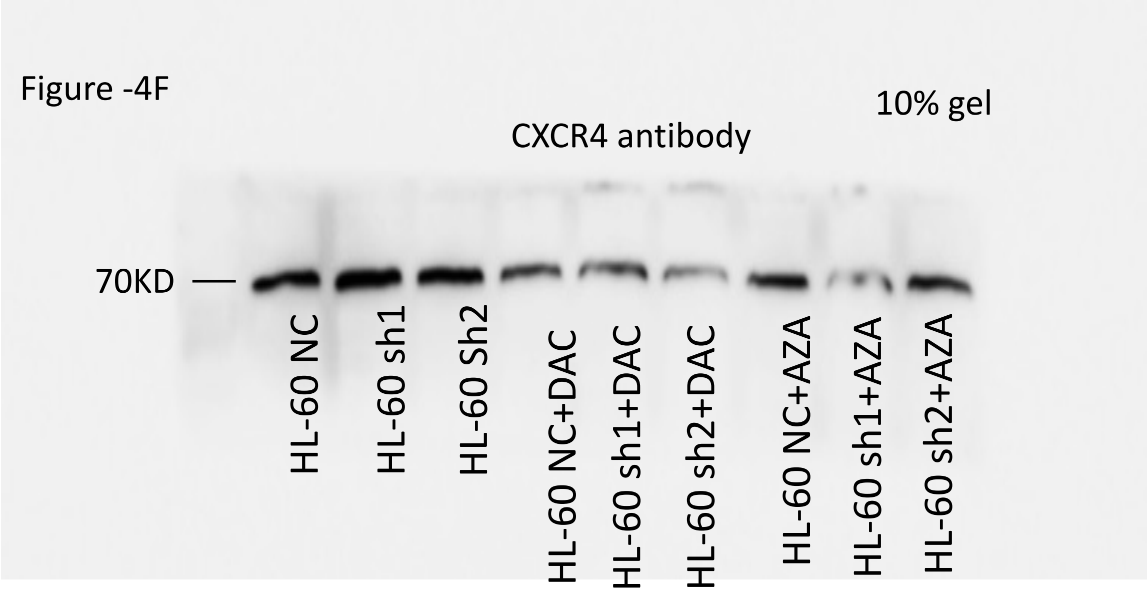
Figure -4F
CXCR4 antibody
10% gel
70KD
HL-60 NC
HL-60 sh1
HL-60 Sh2
HL-60 NC+DAC
HL-60 sh1+DAC
HL-60 sh2+DAC
HL-60 NC+AZA
HL-60 sh1+AZA
HL-60 sh2+AZA

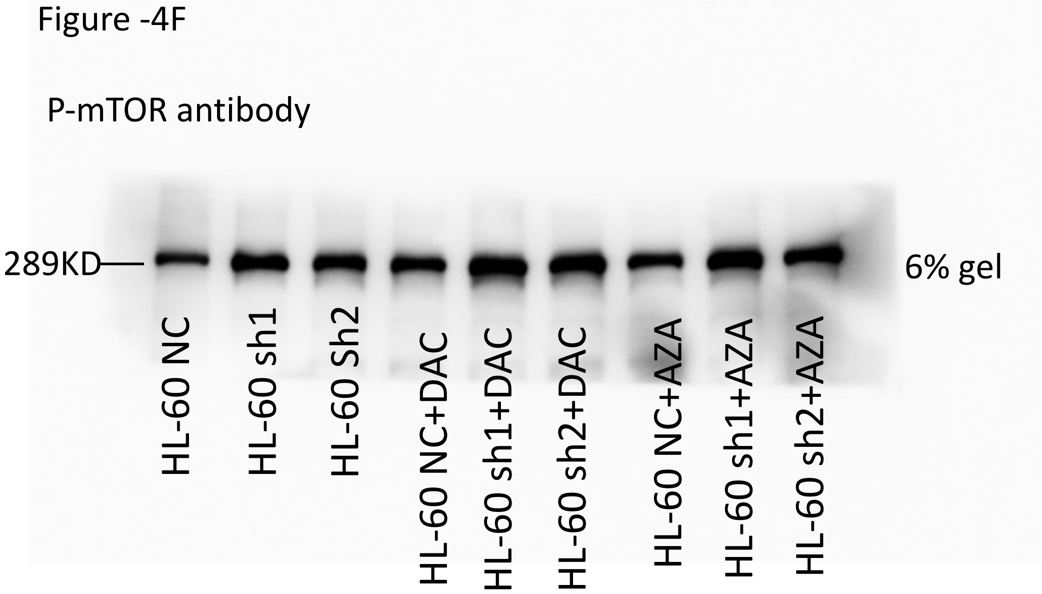
Figure -4F
P-mTOR antibody
289KD
6% gel
HL-60 NC
HL-60 sh1
HL-60 Sh2
HL-60 NC+DAC
HL-60 sh1+DAC
HL-60 sh2+DAC
HL-60 NC+AZA
HL-60 sh1+AZA
HL-60 sh2+AZA

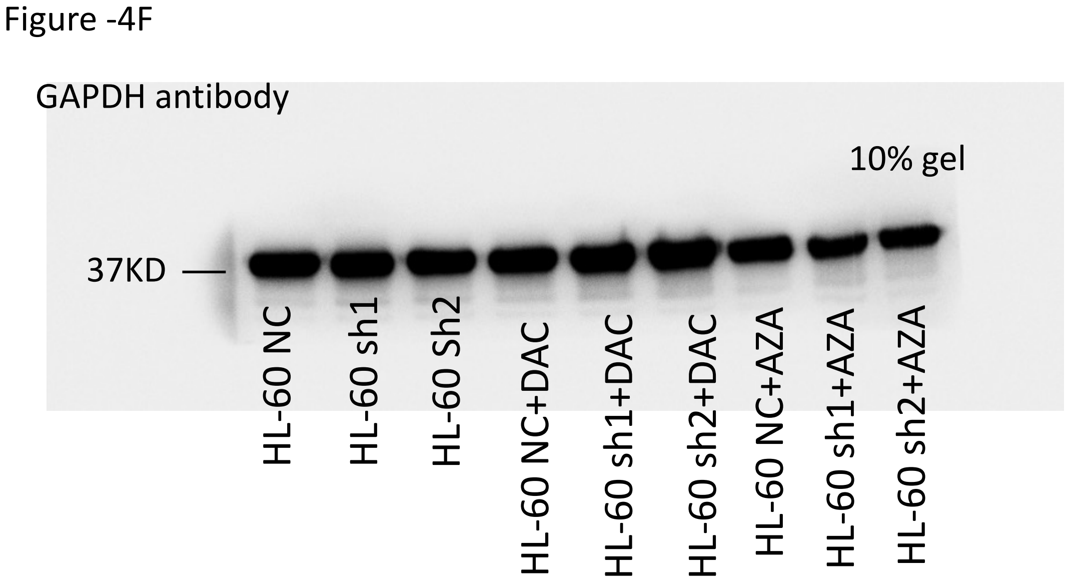
Figure -4F
GAPDH antibody
10% gel
37KD
HL-60 NC
HL-60 sh1
HL-60 Sh2
HL-60 NC+DAC
HL-60 sh1+DAC
HL-60 sh2+DAC
HL-60 NC+AZA
HL-60 sh1+AZA
HL-60 sh2+AZA

Figure -4I

mTOR antibody

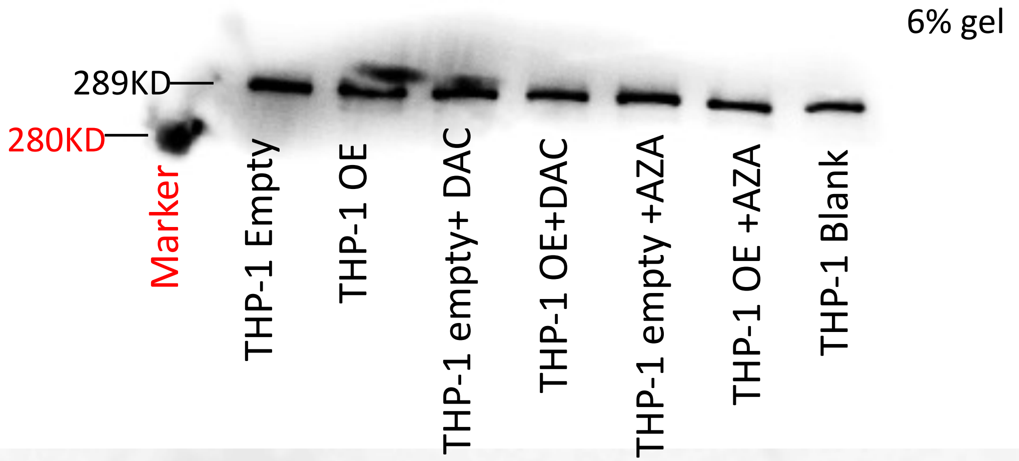

Figure -4I

CXCR4 antibody

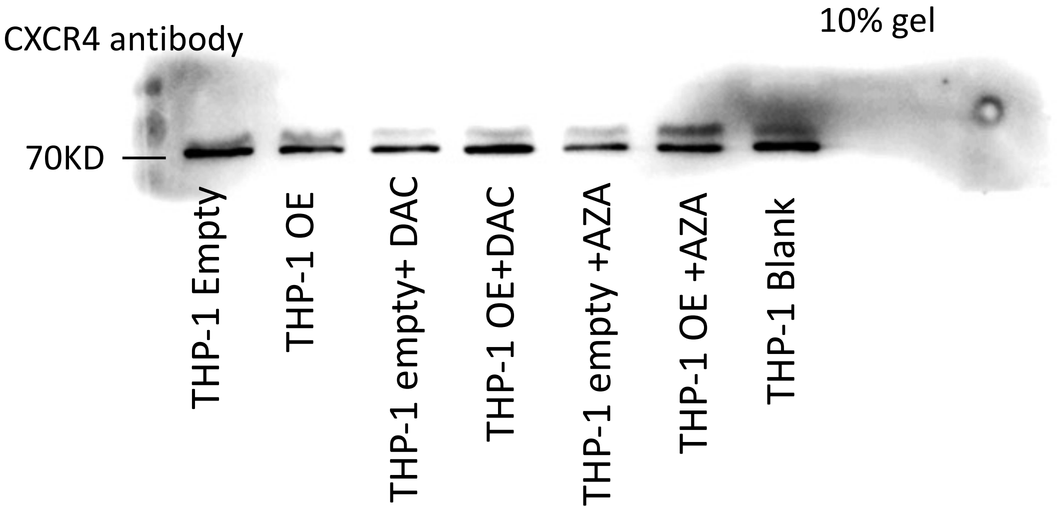

Figure -4I

P-mTOR antibody

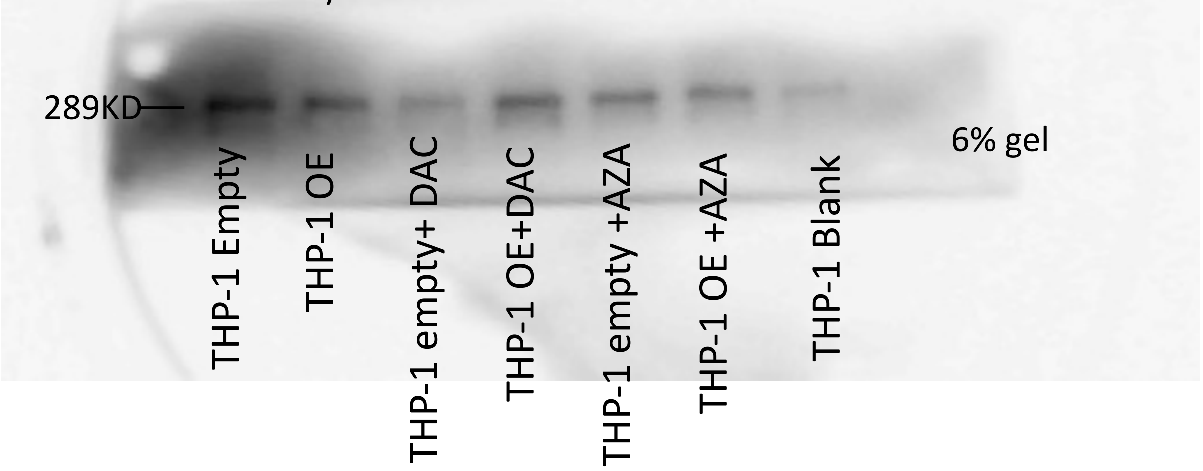

Figure -4I

GAPDH antibody

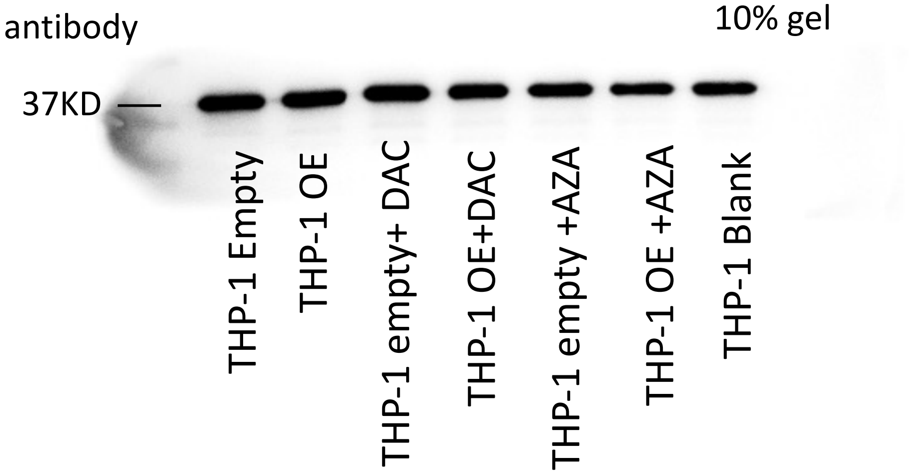

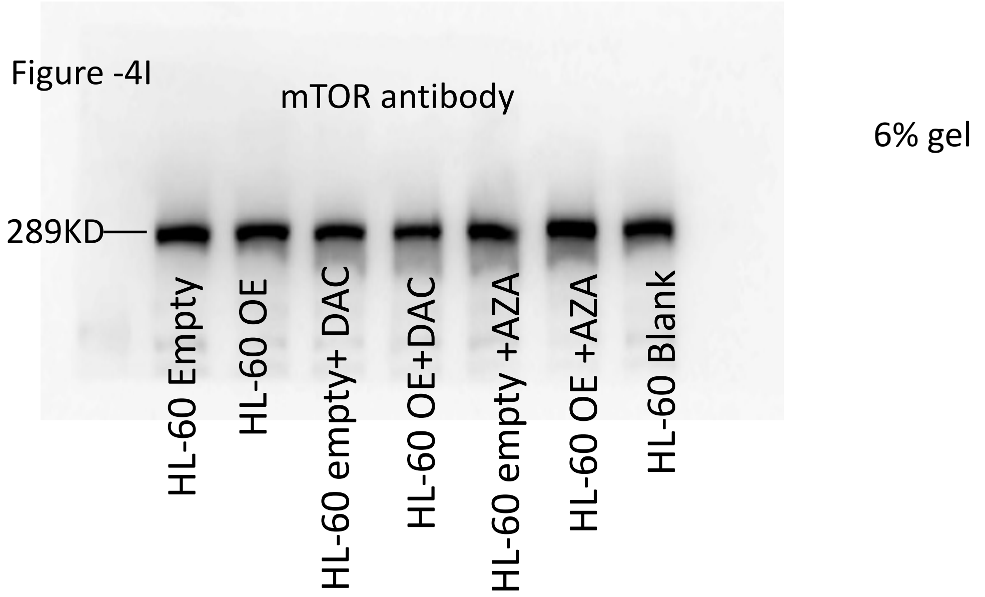
Figure -4I
mTOR antibody
6% gel
289KD
HL-60 Empty
HL-60 OE
HL-60 empty+ DAC
HL-60 OE+DAC
HL-60 empty +AZA
HL-60 OE +AZA
HL-60 Blank

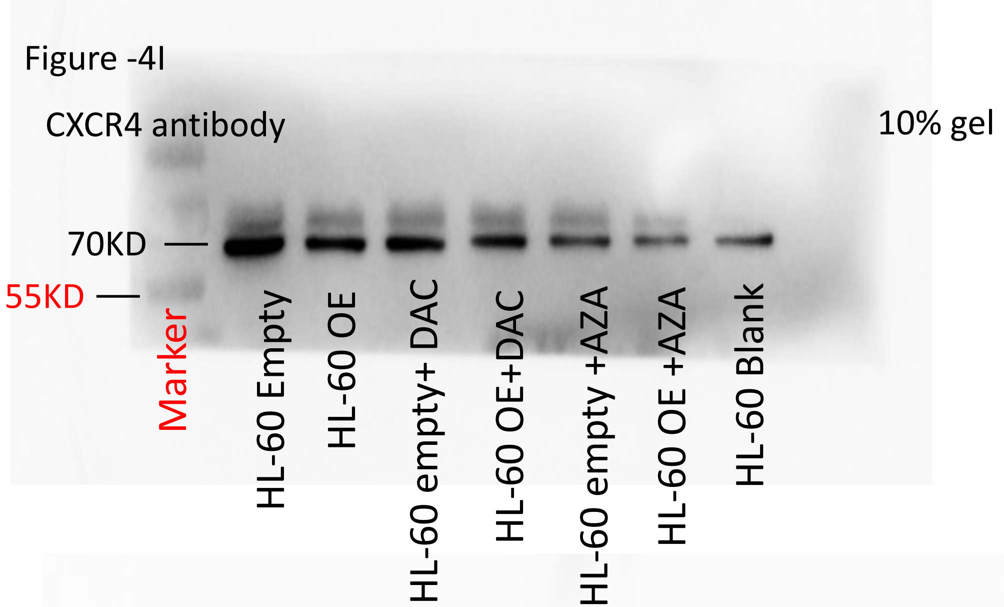
Figure -4I
CXCR4 antibody
10% gel
70KD
55KD
Marker
HL-60 Empty
HL-60 OE
HL-60 empty+ DAC
HL-60 OE+DAC
HL-60 empty +AZA
HL-60 OE +AZA
HL-60 Blank

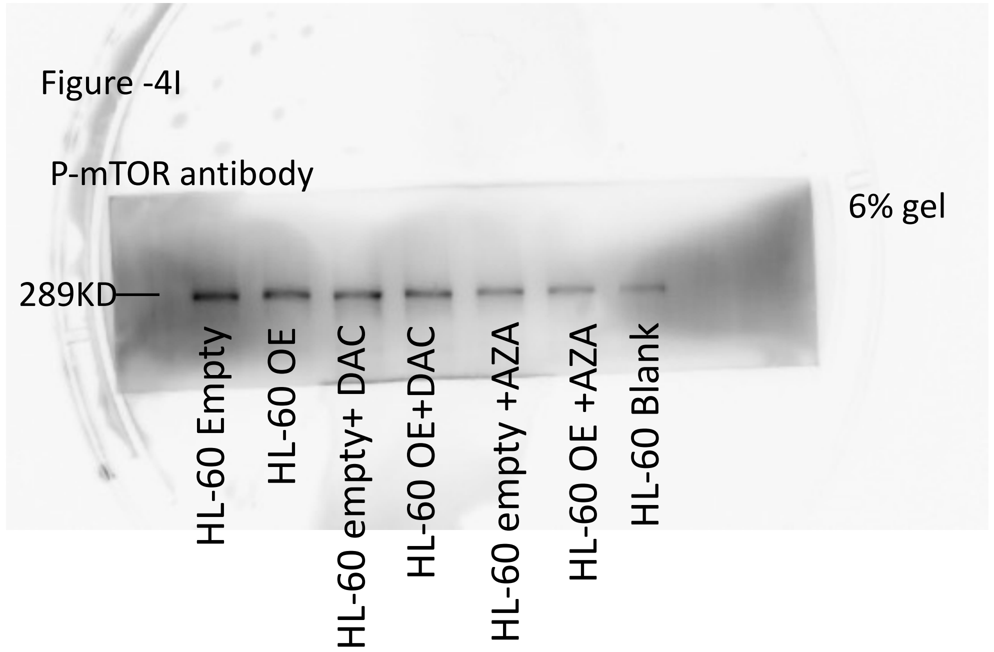
Figure -4I
P-mTOR antibody
6% gel
289KD
HL-60 Empty
HL-60 OE
HL-60 empty+ DAC
HL-60 OE+DAC
HL-60 empty +AZA
HL-60 OE +AZA
HL-60 Blank

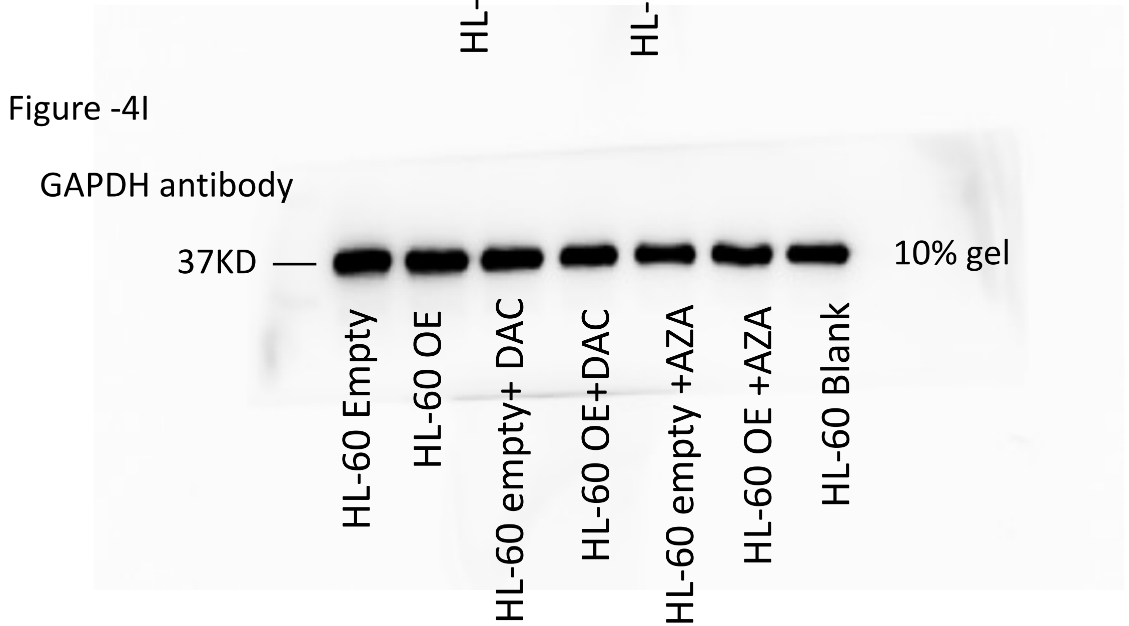
Figure -4I
GAPDH antibody
37KD
10% gel
HL-60 Empty
HL-60 OE
HL-60 empty+ DAC
HL-60 OE+DAC
HL-60 empty +AZA
HL-60 OE +AZA
HL-60 Blank

Figure -5B and 5H

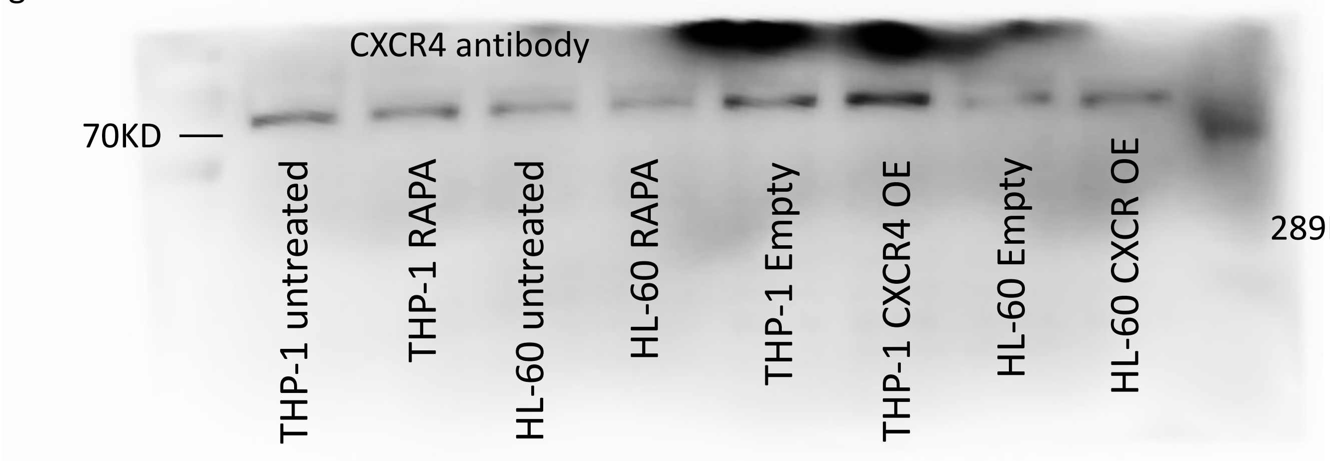

Figure -5B and 5H

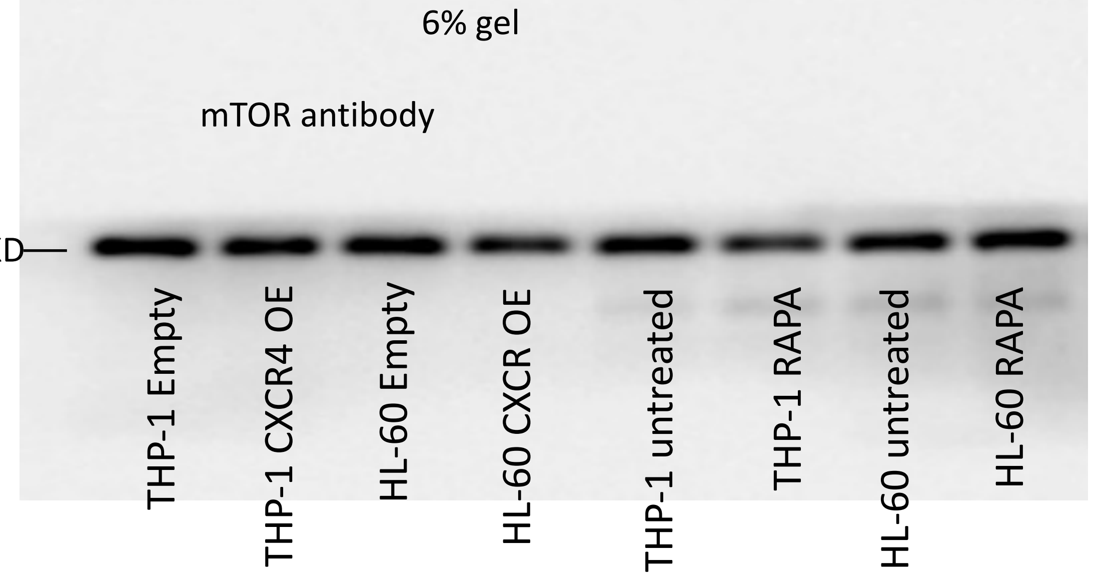

Figure -5B and 5H

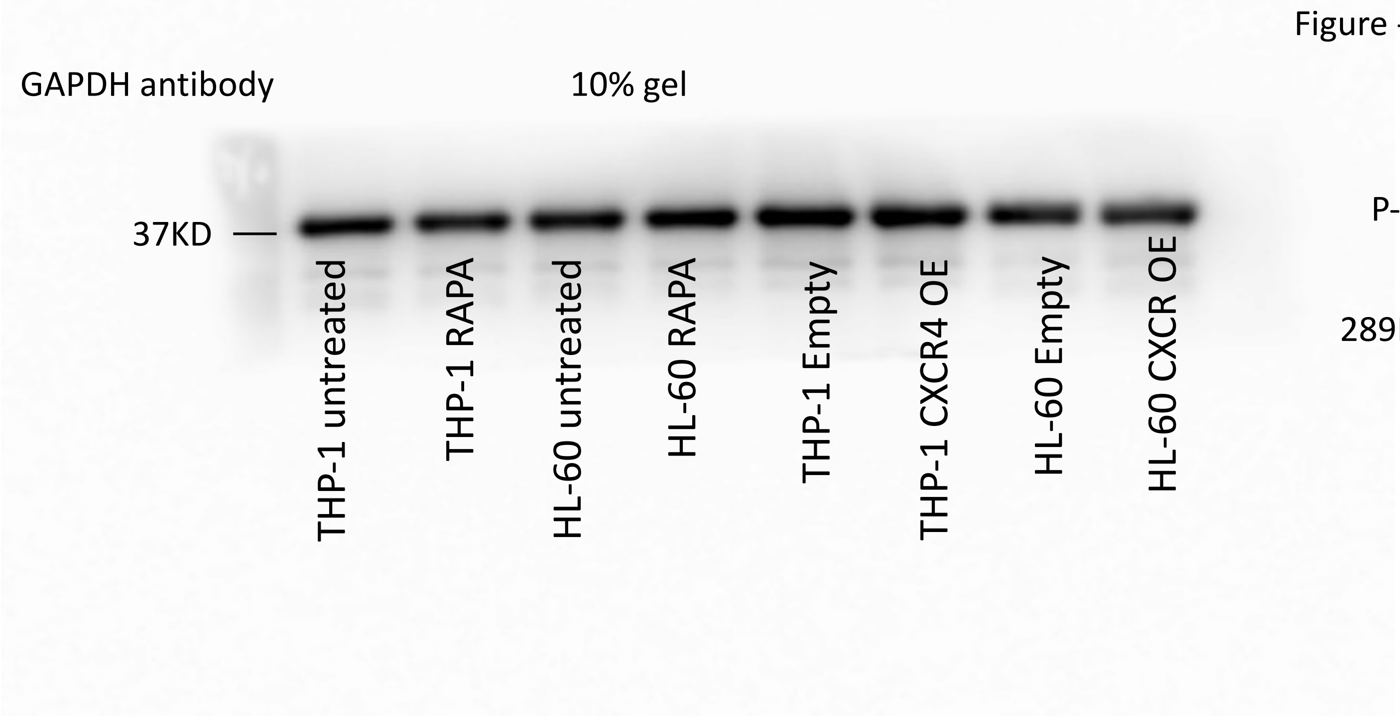

Figure -5B and 5H

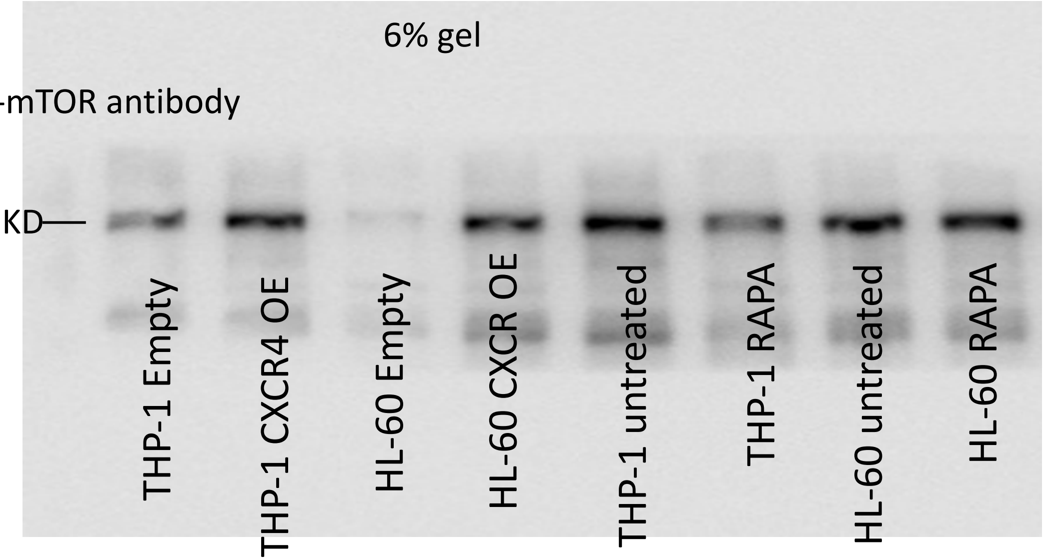

Supplement: Supplementary file 2 — Supplementary Original WB [file 41420_2022_1170_MOESM2_ESM.pdf]
